# P-2129. Analytical Validation of a Next-Generation Sequencing Assay for the Detection of Microbial Pathogens in Human Plasma

**DOI:** 10.1093/ofid/ofae631.2285

**Published:** 2025-01-29

**Authors:** Curtis Bacon, Ellis Bixler, Scott Cowden, Jamie Nutt, Steve Kleiboeker

**Affiliations:** Eurofins Viracor, Lenexa, Kansas; Eurofins Viracor, Lenexa, Kansas; Eurofins Viracor, Lenexa, Kansas; Eurofins Viracor, Lenexa, Kansas; Eurofins Viracor, Lenexa, Kansas

## Abstract

**Background:**

Fungal and acid-fast bacteria (AFB) infections can be a serious complication for immunosuppressed patients. Identification of the pathogen involved in invasive fungal/AFB infections (IFIs) can allow for reduced time to initiation of the appropriate targeted therapy through elimination of ineffective therapies. Current fungal/AFB diagnostics suffer from low sensitivity/specificity, invasiveness (e.g., biopsy or antigen), have long turnaround times (e.g., culture), and/or require an *a priori* hypothesis regarding the causative pathogen (e.g., PCR based methods). To overcome shortcomings in fungal/AFB diagnostics, an unbiased next-generation sequencing (NGS) approach can be utilized to characterize pathogen cfDNA present in a patient blood sample.

**Methods:**

Sequencing of IFIs through hybrid capture has the potential to detect a broad range of pathogens yet can still specifically target the most clinically relevant. Compared to standard whole-genome sequencing methods, hybrid capture followed by NGS can be more specific and sensitive due to an enrichment step using probes specific to cfDNA targets of interest. This additional enrichment step can result in more sequencing reads being dedicated to cfDNA derived from pathogens of interest rather than to cfDNA derived from non-relevant organisms. Here is described a hybrid capture assay that uses >30,000 probes to detect >600 Fungal and select AFB species in plasma.

**Results:**

Cutoffs for target coverage and kmers/million were established through ROC analysis of 170 contrived plasma samples resulting in an analytical specificity of ∼96% and a sensitivity of ∼1,200 molecules/mL. 98% of the 40 contrived samples used for accuracy were correctly identified while 100% intra- and 93%-inter assay precision was achieved. *In silico* validation demonstrated the assay’s ability to detect closely related species unable to be tested at the bench. Several species have been detected in the initial clinical samples received thus far, however, all have been below the reportable cutoff values.

**Conclusion:**

The analytical validation described here demonstrates the analytical performance of hybrid capture in human plasma.

**Disclosures:**

Curtis Bacon, PhD, Eurofins Viracor: employee Ellis Bixler, n/a, Eurofins Viracor: Employee Scott Cowden, n/a, Eurofins Viracor: Employee Jamie Nutt, n/a, Eurofins Viracor: Employee Steve Kleiboeker, PhD, Eurofins: employee|Eurofins: Stocks/Bonds (Public Company)

